# *Moniezia benedeni* infection promoting ICOS^+^ T cell proliferation in sheep (Ovis aries) small intestine

**DOI:** 10.1186/s12917-025-04761-5

**Published:** 2025-05-03

**Authors:** Baoshan Wang, Wanling Yao, LiLan Zhang, Lidong Jiang, Jing Pan, Wenzhu Chai, Zhen Huang, Sihan Zuo, Zhenpeng Li, Yanming Wei, Wangdong Zhang

**Affiliations:** College of Veterinary Medicine, Gansu Agricultural University, Lanzhou, 730070 China

**Keywords:** Sheep, Small intestine, *Moniezia benedeni* infection, ICOS^+^ T cells, Prokaryotic expression

## Abstract

**Background:**

Cellular immunity mechanisms play a crucial role in regulating anti-parasite immunity. ICOS is one of the core factors of multitype T cell subsets involved in the regulation of immune homeostasis. The aim of this experiment was to investigate the distribution patterns of ICOS^+^ T cells in the small intestine of sheep and determine the impact of *Moniezia benedeni* (*M. benedeni*) infection on these cells.

**Methods:**

In this study, a sheep pET-28a-ICOS recombinant plasmid was constructed, and the recombinant protein was obtained through induced expression in BL21 (DE3) cells. Furthermore, a rabbit polyclonal antibody against sheep ICOS was produced. The expression of ICOS in the sheep small intestine was analyzed using immunofluorescence and ELISA, comparing the results before and after *M. benedeni* infection.

**Results:**

The findings revealed that the purified recombinant ICOS protein had the anticipated size (14.2 kDa). The rabbit anti-sheep ICOS polyclonal antibody showed good specificity and a titer of 1:128,000. ELISA results indicated a significant increase in ICOS expression in all segments of the small intestine after *M. benedeni* infection (*P* < *0.05*). The ileum exhibited the most substantial increase in expression (*P* < *0.001*), followed by the jejunum (*P* < *0.05*) and duodenum (*P* < *0.05*). Immunofluorescence analysis demonstrated that ICOS^+^ T cells are diffusely distributed in the intestinal epithelium and around the intestinal glands in the lamina propria of the duodenum, jejunum, and ileum of sheep. Moreover, after being infected with *M. benedeni*, the number of ICOS^+^ T cells in all intestinal segments significantly increases (*P* < *0.05*), with the most significant increase in the intestinal epithelium of the duodenum.

**Conclusions:**

These findings suggest that *M. benedeni* infection in sheep can stimulate the proliferation of ICOS^+^ T cells in the small intestine. This lays the foundation for future research on the role of ICOS^+^ T cells in regulating cellular immunity against parasitic infections in different segments of the small intestine.

**Graphical Abstract:**

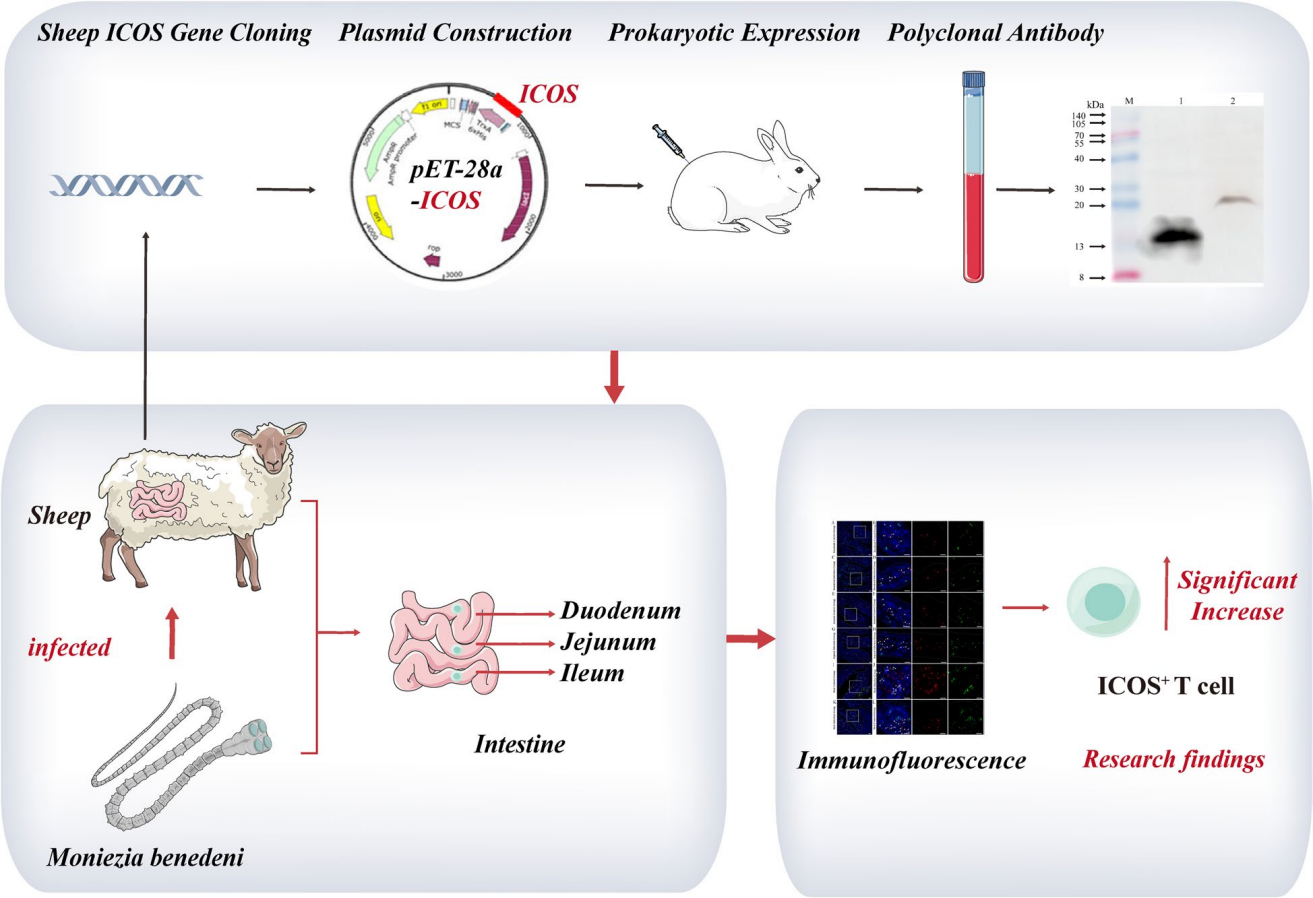

**Supplementary Information:**

The online version contains supplementary material available at 10.1186/s12917-025-04761-5.

## Background

The inducible T-cell costimulator (ICOS), also known as CD278, is composed of an extracellular immunoglobulin domain, a transmembrane region and a cytoplasmic part, and is a type I transmembrane glycoprotein [1]. It is an important member of the CD28 family, which has been identified the thirdly [2]. It was found that ICOS was highly expressed on T cells in the tonsils, and was closely related to B cells in the apical bright zone of the germinal center, the terminal region where B cell mature can induce B cells to differentiate into antibody-secreting cells or memory cells [2]. It has been demonstrated that ICOS is expressed on multiple T cell subsets, including Th1 [3], Th2 [4], Th17 [3, 5, 6], Th22 [7], Tfh [8], and Treg [9] cells. Unlike CD28 and CTLA-4, ICOS lacks a specific MYPPPY motifs. Therefore, the signaling of ICOS is not a transduction process completed by interaction with CD80 and CD86 but is exerted through binding to its specific ligand ICOSL [10]. ICOSL is expressed not only on immune cells, such as dendritic cells [11, 12], B-cells [13–15], plasma cells [16], mast cells [17], eosinophils [18], macrophages [19], and ILC2s [20], but also on somatic cells, such as endothelial cells [4], lung epithelial cells [21], muscle cells [22], bone marrow mesenchymal stem cells [23] and tumor cells [24], etc. Therefore, ICOS plays an important role not only in regulating the activation process of T cells and initiating the process of humoral immunity but also in the regulation of homeostasis in various organs. For example, it was reported that ICOS^−/−^ mice were more susceptible to bacterial (such as *M.tuberculosis* [25], *C.muridarum* [26], and *S.enterica* [27], etc.) and parasitic (such as *S.mansoni* [28], *T.gondii* [29], and *T.spiralis* [30], etc.) infections, and the Th1 response was weakened and the pathological changes caused by pathogens were aggravated. It was also found that ICOS^−/−^ mice had a decrease in Treg cells during *H.polygyrus* infection [31], and had a decrease in Th2 cells but had an increase in parasite egg production during *N.brasiliensis* infection [32]. ICOS is expressed on subpopulations of Th2 cells and plays a crucial role. Studies found that blocking ICOS signaling could hinder the differentiation of CD4^+^ T cells into Th2 and reduce the production of IL-4, IL-5, and IgE [30]. During *T.spiralis* infection, the production of TNF-α, IFN-γ and IL-10 was promoted [30]. In summary, ICOS plays a crucial role in regulating the proliferation, survival, and differentiation of T cells, and it is great significance for the effectiveness of the immune responses and the formation of immune memory. Similarly, ICOS can also play a crucial role in anti-parasite infection immunity by regulating the immune response of Th cells.

At present, there is few reports on the mechanism of ICOS inducing the host to produce anti-parasitic infection in *M. benedeni* infection. The present study would perform the bioinformatics analysis of the ICOS gene, prokaryotic expression, polyclonal antibody production, and thereby systematically analyzing the distribution of ICOS^+^ T cells and the expression characteristics of ICOS in the small intestine of sheep infected with *M. benedeni*. This present study will provide a foundation for further research on the molecular mechanism of the mucosal immune network of the digestive tract of sheep in response to parasite parasitism.

## Materials and methods

### Experimental animals and experimental design

Control male New Zealand large white rabbits weighing approximately 2.5 kg were purchased from the Laboratory Animal Centre, Lanzhou Institute of Veterinary Medicine, Chinese Academy of Agricultural Sciences. The rabbits were anesthetized by intravenous injection of sodium pentobarbital (30 mg/kg) via the ear vein, fixed in a supine position on the operating platform under moderate anesthesia, and blood was aseptically collected from the heart. The collected blood was stored in a 4 °C refrigerator for 12 h to allow the serum to separate, and then the supernatant serum was collected and stored in a in a -80 °C refrigerator for future use.

The sheep we collected were all 8–10 months old, female, and naturally infected. The collection time was from July to September (this period was the high incidence period of local tapeworm infection); without deworming, the parasitic time of tapeworms in sheep was about 6–8 months, which could effectively rule out the possibility of repeated infection. During the collection of clinical samples, whether there was tapeworm infection was determined based on whether there were mature gravid proglottids in the feces, and fecal egg detection was conducted simultaneously to exclude individuals with mixed parasite infections and determine naturally infected individuals; in the same group, individuals of the same gender and age that were not infected with *M. benedeni* were selected as the control group at the same time. All sheep are quarantined from Wenkui Slaughterhouse, Liangzhou District, Wuwei City, Gansu Province.

Six uninfected (Control group, *n* = 6) and 6 sheep infected with *M. benedeni* (Infected group, *n* = 6) naturally were selected, respectively. The sheep were anesthetized by intravenous injection of 20 mg/kg sodium pentobarbital and then euthanized. After dissecting the abdomen, we separately took the tissues samples from the duodenum, jejunum, and ileum. The samples were quickly frozen in liquid nitrogen and stored in a refrigerator at -80 °C for ELISA and Western blotting experiments. Histological specimens were fixed in 4% neutral paraformaldehyde solution for 20 days, then dehydrated, embedded and sectioned into 4 μm slices using conventional methods for immunofluorescence experiments.

### Bioinformatics analysis of the sheep ICOS gene

According to the coding region (CDS) region of ICOS nucleotide sequences of various species obtained from NCBI database, *Ovis aries*, *Homo sapiens*, *Canis lupus familiaris*, *Equus caballus*, *Bos taurus*, *Sus scrofa*, *Panthera pardus*, *Camelus dromedarius*, *Rhinopithecus roxellana*, *Vicugna pacos*, *Capra hircus*, *Gallus gallus*, *Oryctolagus cuniculus* and *Mus musculus*. MAGA 11.0 software was used for homology comparison and phylogenetic tree construction (Additional file 1). Some online analysis software were used to predict and analyze the basic physicochemical properties of sheep ICOS genes, protein hydrophilicity/hydrophobicity, transmembrane structure, signal peptides, subcellular localization, secondary and tertiary structures, phosphorylation and glycosylation sites, and protein interactions, respectively (Additional file 2).

### Synthesis of sheep ICOS gene and construction of recombinant plasmids

Referring to the coding region (CDS) of the sheep ICOS gene sequence recorded in NCBI, it consists of 209 amino acids. According to the analysis, amino acids 1–142 make up the extramembrane portion, 1–19 are the signal peptide portion, which was truncated, leaving 123 amino acids. The nucleotide sequence of this portion was modified to add the enzymatic cleavage sites 5’*BamH*I and 3’*Xho*I (Additional file 3). The modified nucleotide sequence was optimized and synthesized by Jin Wei Zhi Biotechnology Co. After ligation with the pET-28a( +) vector, it was transformed into DH5α competent cells, and the recombinant plasmid was named pET-28a-ICOS.

### Prokaryotic expression and antibody preparation of pET-28a-ICOS recombinant plasmid

The plasmid pET-28a-ICOS was transferred into BL21 (ED3) competent cells (Solarbio Biotechnology Co., Ltd.) and single colony was picked from an LB medium containing 1 mmol/L kanamycin. After induction with 1 mmol/L IPTG for 6 h, the cells were collected, and SDS-PAGE was used to detect and analyze the expression of induced protein. The ICOS recombinant protein was prepared using a His-tagged protein purification kit. The protein content was determined using a spectrophotometer, and the purity of the recombinant protein was detected using SDS-PAGE. The purified ICOS recombinant protein was mixed with adjuvant and fully emulsified. Then the rabbits were immunized with 800 μg/rabbit by multiple subcutaneous injections at the back for the first immunization, followed by the second and third immunizations after 1 and 2 weeks, respectively, with the same dosage. The blood was collected from the heart after 3 weeks to obtain polyclonal rabbit anti-sheep ICOS serum.

### Detection of rabbit anti-sheep ICOS serum titer and Western blotting analysis

The ICOS recombinant protein was used as antigen and coated onto the wells of an enzyme-linked immunosorbent assay (ELISA) plate at 5 μg/well and incubated overnight at 4 °C. The antiserum was diluted in a gradient of 1:2000–1:128000, and after washing, and then the HRP-labelled goat anti-rabbit secondary antibody was added. After incubation for 1 h, the H_2_SO_4_ was added to stop the reaction and the TMB substrate chromogenic solution was added to develop color in the dark conditions. The absorbance value at 450 nm was measured with a microplate reader. The highest dilution of serum corresponding to D450 _nm (positive)_/D450 _nm (negative)_ ≥ 2.1 was considered as the titer of the polyclonal serum.

ICOS recombinant and natural proteins were separated on a 15% SDS-PAGE gels and transferred onto PVDF membranes. The membranes were blocked in 5% skim milk at room temperature for 2 h, and then incubated with rabbit antiserum as the primary antibody (1: 1000 dilution) overnight at 4 °C. After washing with PBS, the membrane was incubated with HRP-labelled secondary antibody (1: 5000 dilution) for 2 h at room temperature, and the color was developed using ECL luminescent solution.

### Detection of ICOS expression level

The frozen tissues were thawed on ice and 1.0 g was accurately weighed and placed in a 2 ml EP tube. The tissue was homogenized at -10 °C for 20 min, then centrifuged for 10 min (4 °C, 12,000 rpm), and the supernatant was collected. The protein concentration was determined using the BCA method (BCA Protein Assay Kit, Cat#PC0020, Lot). The sheep ICOS ELISA KIT (Shanghai Enzyme Linked Biotechnology Co., LTD) was used to detect the content of ICOS in each segment of intestinal tissue.

### Double immunofluorescence staining for ICOS^+^ T cells

Paraffin sections (4 μm) were prepared from formaldehyde-fixed specimens by routine method. The CD3 and ICOS immunofluorescence double labeling experiments were performed using the Dual-Color Immunofluorescence Kit (RC0086-23RM, Shanghai Rutron). The CD3 antibody was diluted 1: 600 [33], and the ICOS antibody was diluted 1:500. The distribution characteristics of ICOS^+^ T cells in the sheep small intestine were observed using a fluorescence microscope (The DV Elite™Imaging System, GE, USA), and images were captured. Three slices of each part (including duodenum, jejunum, and ileum) of each sample in the Infected and Control groups were observed, 12 fields of view from each tissue slices were selected to perform optical density analysis to quantify CD3 and ICOS using Image J software.

### Statistical analysis

Statistical analysis was carried out using SPSS 23.0 (SPSS Inc, Chicago, USA). One-way ANOVA was used to analyze differences among sites within the same group, and the independent t-test differences was used to analyze the differences between the infected and control groups for the same part in were analyzed separately using for independence. of *P* < *0.05* was considered statistically significant.

## Results

### Sequence similarity comparison and phylogenetic tree construction of sheep ICOS gene

**Fig. 1:**
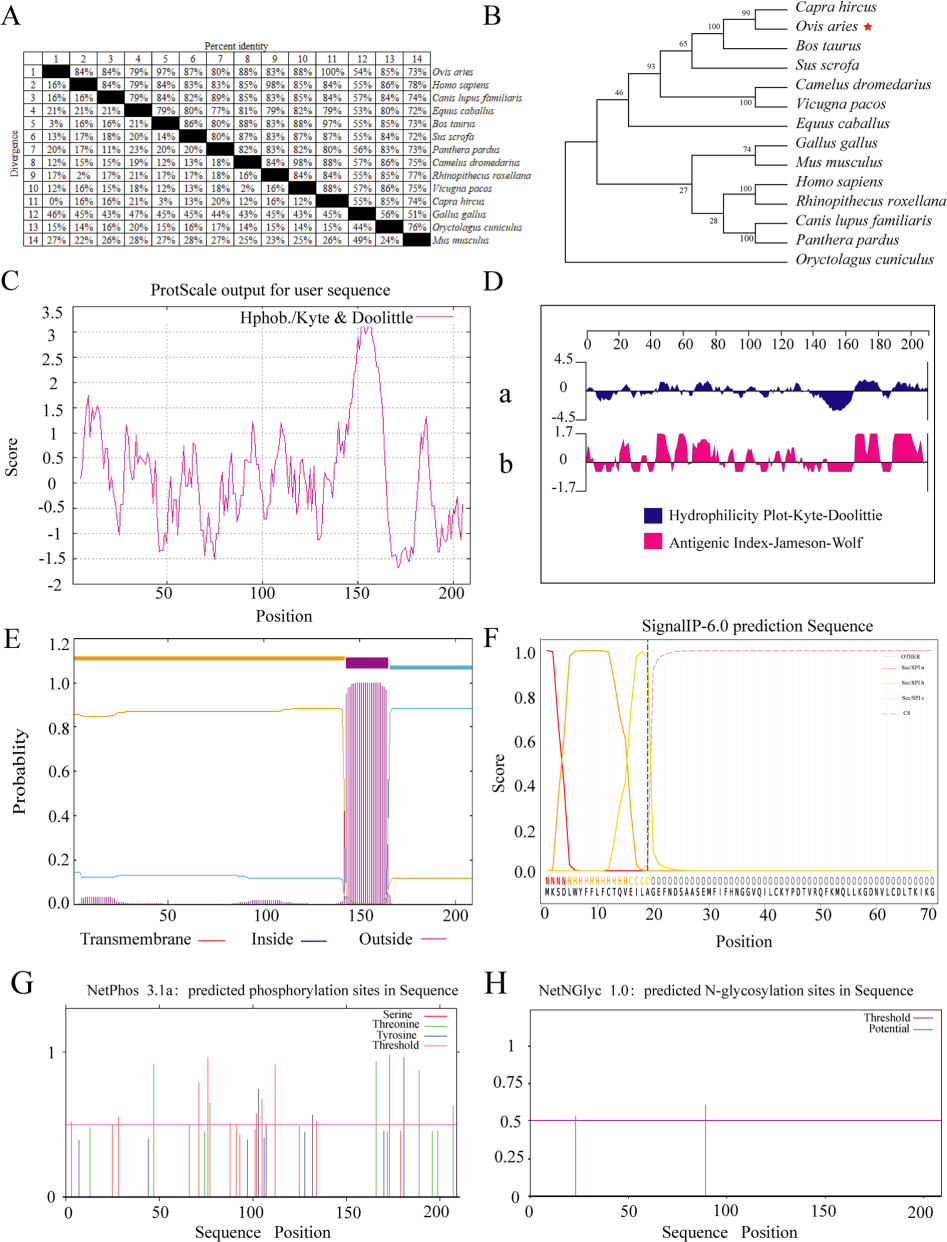
Bioinformatics prediction of sheep ICOS gene. **A** Similarity comparison of sheep ICOS gene. **B** Phylogenetic tree construction of sheep ICOS gene; **C** Hydrophilicity /hydrophobicity prediction of sheep ICOS protein; **D** Hydrophilicity and antigenic epitope prediction of sheep ICOS protein (a. Hydrophilicity prediction; b. Antigenic epitope prediction); **E** Transmembrane region prediction of sheep ICOS protein (red line indicates transmembrane region; blue line indicates intramembrane region; purple line Indicates extramembrane region); **F** Signal peptide prediction of sheep ICOS protein (Sec/SPI n. N-terminal region of signal peptide; Sec/SPI h. Central hydrophobic region of the signal peptide; Sec/SPI c. C-terminal region of signal peptide). **G** Prediction of potential phosphorylation sites of sheep ICOS protein. **H** Prediction of potential glycosylation sites of sheep ICOS protein

The similarity of the coding region CDS regions of the ICOS gene sequences between *Ovis aries* and *Homo sapiens*, *Canis lupus familiaris*, *Equus caballus*, *Bos taurus*, *Sus scrofa*, *Panthera pardus*, *Camelus dromedarius*, *Rhinopithecus roxellana*, *Vicugna pacos*, *Capra hircus*, *Gallus gallus*, *Oryctolagus cuniculus* and *Mus musculus* were 97%, 88%, 88%, 87%, 85%, 84%, 84%, 83%, 80%, 79%, 73%, 54%, and 100%, respectively (Fig. 1A). The evolutionary tree analysis indicates that *Ovis aries* is most closely related to *Capra hircus*, followed by *Bos taurus* and *Camelus dromedarius*, with the most distant relationship to *Gallus gallus* (Fig. 1B).

### Physicochemical properties of sheep ICOS protein

The ICOS protein is composed of 209 amino acids with a molecular weight of 23,852.71 Da. Its molecular formula is C_1092_H_1659_N_261_O_306_S_16_. The protein is acidic with a theoretical isoelectric point (pI) of 6.11. The predicted instability coefficient is 34.43, suggesting that the ICOS protein is stable. As shown in Table 1, among the 22 amino acids encoding ICOS, leucine is the most abundant with 23 residues (Accounting for 11.00%); while histidine is the least abundant with only 2 residues (Accounting for 1.00%). There is no tryptophan, pyrrolysine, and selenocysteine, indicating that the ICOS protein has no enzymatic activity. A total of 37 serine (Ser), tyrosine (Tyr), and threonine (Thr) residues, accounting for 17.80%, suggesting that ICOS protein is susceptible to phosphorylation modification.

**Table 1:** Amino acid composition of sheep ICOS protein

Amino acids	Number	Proportion	Amino acids	Number	Proportion
Ala(A)	11	5.30%	Lys(K)	15	7.20%
Arg(R)	3	1.40%	Met(M)	6	2.90%
Asn(N)	12	5.70%	Phe(F)	17	8.10%
Asp(D)	11	5.30%	Pro(P)	10	4.80%
Cys(C)	10	4.80%	Ser(S)	16	7.70%
Gln(Q)	9	4.30%	Thr(T)	11	5.30%
Glu(E)	8	3.80%	Trp(W)	3	1.40%
Gly(G)	8	3.80%	Tyr(Y)	10	4.80%
His(H)	2	1.00%	Val(V)	14	6.70%
Ile(I)	10	4.80%	Pyl(O)	0	0.00%
Leu(L)	23	11.00%	Sec(U)	0	0.00%

### Prediction of hydrophilicity/hydrophobicity, transmembrane regions, and signal peptides of sheep ICOS protein

The hydrophilicity/hydrophobicity prediction of amino acid sequence of sheep ICOS protein showed that the highest value was at amino acid 153 (isoleucine), with a score of 3.200, indicating the strongest hydrophobicity. The lowest value was at amino acid 171 (proline), with a score of -1.678, indicating the strongest hydrophilicity (Fig. 1C). The hydrophilic region contained a higher proportion of amino acids than the hydrophobic region, suggesting that the sheep ICOS protein was hydrophilic. The prediction analysis of hydrophilicity (Fig. 1Da) and antigenic epitope (Fig. 1Db) of sheep ICOS protein also indicated that it was a hydrophilic protein with a high antigenic index. The sheep ICOS protein was predicated to have the following domains: positions 1–142 belonged to the extramembrane region, positions 143–165 were within the transmembrane region, and positions 166–209 were in the intramembrane region (Fig. 1E). Signal peptide prediction showed that this protein had a Tat signal peptide (Tat/SPI) and the that the cleavage site was likely to be between position 19 and 20 with a probability of 0.973042 (Fig. 1F).

### Subcellular localization prediction of sheep ICOS protein

Sheep ICOS protein is predicted to be localized in the cell membrane, cytoplasm, extracellular space, mitochondria, and nucleus. Therefore, it can be inferred that sheep ICOS performs its main biological function as a membrane protein.

### Prediction of the secondary and tertiary structure of sheep ICOS protein

**Fig. 2:**
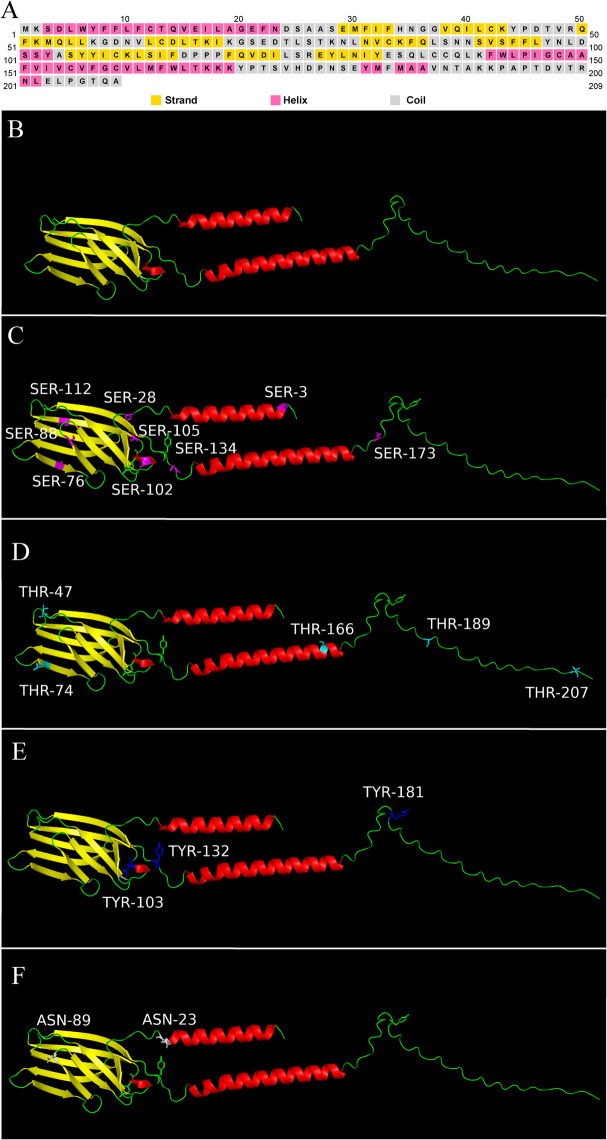
Prediction of secondary and tertiary structure of sheep ICOS protein and its potential phosphorylation and glycosylation sites. **A** Secondary structure of ICOS (red: α-helices; yellow: β-turns; green: irregular curls); **B** Tertiary structure of ICOS; **C** Position of serine in the tertiary structure; **D** Position of lysine in the tertiary structure; **E **Position of threonine in the tertiary structure; **F** Position of asparagine in the tertiary structure

The secondary structure of ICOS protein has α-helices, β-turns, extended chains and irregular curls, classifying it as a mixed protein (Fig. 2A). The highest proportion of the irregular curls indicates that there is a larger region of binding sites, suggesting that ICOS is a mixed protein with complex biological functions.

The online tool SWISS-MODEL was used to predict the tertiary structure of sheep ICOS protein. The model had a coverage of 94.74%, indicating that the model is reasonably constructed (Fig. 2B). The tertiary structure model prediction result was consistent with the secondary structure.

### Analysis of potential phosphorylation and glycosylation sites of sheep ICOS

Figure 1G and H display the positions of potential phosphorylation and glycosylation sites, respectively, in the tertiary structure of sheep ICOS protein. The phosphorylation results indicated that there are 10 serines (Fig. 2C), (the tertiary structure prediction model showed only 9 serine sites), 5 tyrosine (Fig. 2D), and 3 threonines (Fig. 2E) that could potentially be phosphorylation sites for the protein kinases (Fig. 2F). Additionally, the glycosylation analysis revealed the presence of 2 asparagines (Fig. 1H) that could potentially be glycosylation sites for the protein kinases (Fig. 2F). Phosphorylation is the primary mode of post-translational modification for this protein.

### Interactions analysis in sheep ICOS protein

**Fig. 3:**
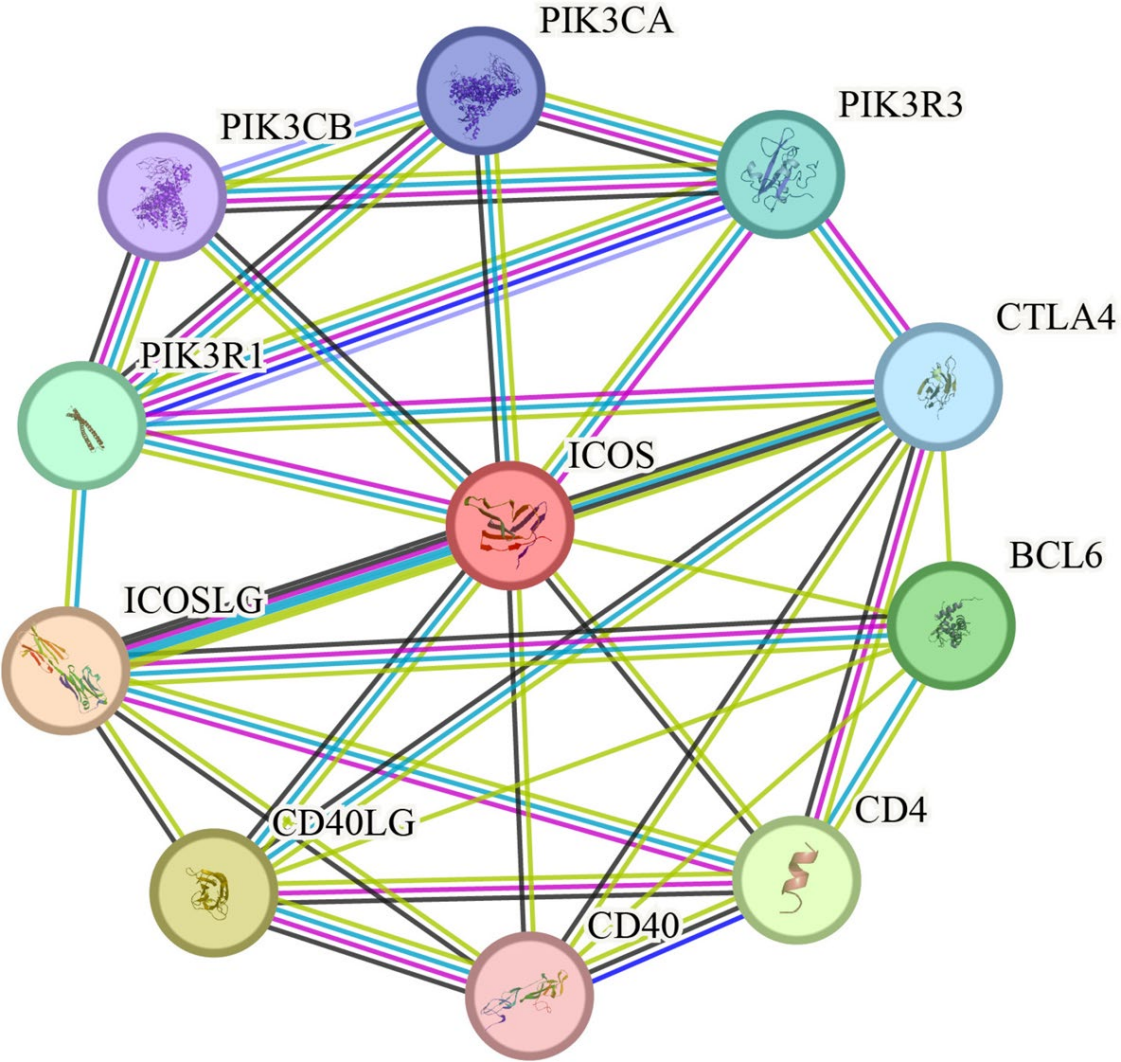
Analysis of protein interactions of sheep ICOS. (Black line is gene co-expression; purple line is experimental/biochemical data showing an association; blue line is an association in a regulatory database; yellow line is common mention in a Pubmed abstract.)

The STRING analysis revealed an average local clustering coefficient of 0.863. The sheep ICOS proteins were predicated to interact with ICOSLG (Inducible T-cell co-stimulator ligand), CD40LG (CD40 ligand), CD4 (T-cell surface glycoprotein CD4 precursor), BCL6 (B cell lymphoma 6), PIK3R1 (Phosphatidylinositol-3-kinase regulatory subunit 1), and PIK3R3 (Phosphatidylinositol-3-kinase regulatory subunit 1). The proteins PIK3R3 (Phosphatidylinositol-3-kinase regulatory subunit 3), CTLA4 (Cytotoxic T-lymphocyte-associated protein 4), PIK3CA (Phosphatidylinositol-4, 5-bisphosphate 3-kinase catalytic subunit alpha), PIK3CB (Phosphatidylinositol-4,5-bisphosphate 3-kinase, catalytic subunit beta), and CD40 (CD40 molecule). And there have strong interconnection among these proteins (Fig. 3).

### Sheep ICOS gene synthesis and construction of recombinant plasmids

The sheep ICOS protein is composed of 209 amino acids with a molecular weight of 23,852.71 Da. The extramembrane portion spans from 1–142, while the signal peptide portion spans from 1–19. After truncating of the signal peptide, 123 amino acids remain. The start codon ATG and the stop codon TAA were added, followed by the enzyme cleavage sites *BamH*I and *Xho*I. After base optimization, the genes were synthesized by Genewiz Biological Technology Co., Ltd. The size of the recombinant protein of ICOS was predicted to be 14.2 kDa. It was then cloned into the pET-28a( +) vector and transformed into BL21 (DE3) competent cells. The constructed recombinant plasmid was named pET-28a-ICOS.

### Induction and expression of pET-28a-ICOS in Escherichia coli

**Fig. 4:**
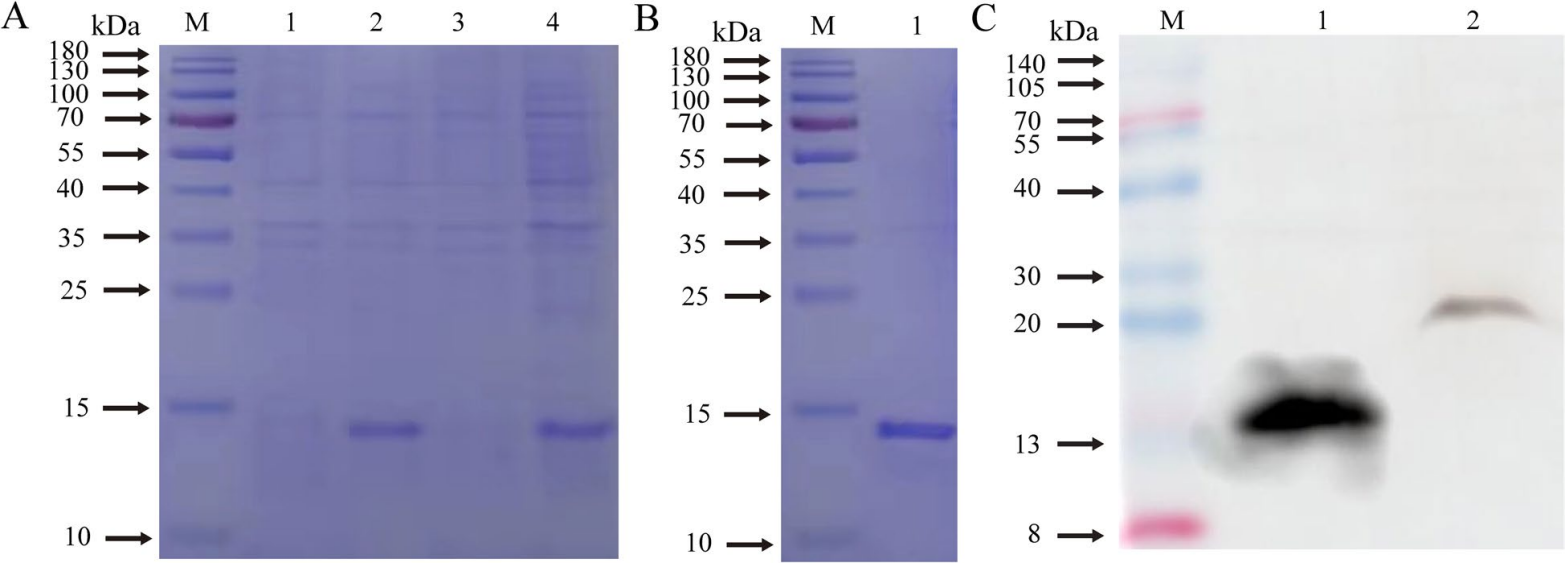
Expression, expression form, detection of purification results and Western blotting identification of recombinant proteins of sheep ICOS. **A** detection of sheep ICOS recombinant protein expression form (M. Protein molecular weight marker; 1. Pre-induction product of recombinant bacteria; 2. Post-induction product of recombinant bacteria; 3. Supernatant of induced product; 4. Precipitation of induced product); **B** Purification results of sheep ICOS recombinant protein (M. Protein molecular weight maker; 1. Purified recombinant protein). **C** Purification results of sheep ICOS recombinant Protein (M. Protein molecular weight marker; 1. Purified recombinant protein; 2. Extracted total natural protein)

The recombinant bacteria were cultured at 37 °C until the OD_600_ nm value reacged 0.8. After 6 h of induction with IPTG (1 mmol/L), the cells were collected and the recombinant pre-induction and post-induction products were detected using SDS-PAGE. Figure 4A shows successful expression of the recombinant protein ICOS in the BL21 (DE3).competent cells (Additional file 4).

### Purification of recombinant sheep ICOS protein

The ICOS recombinant protein was purified using a nickel column. The recombinant protein was purified well without heteroproteins through SDS-PAGE detection, and its size was approximately 14.2 kDa, which was consistent with the predicted size (Fig. 4B). The purified protein content was determined to be 2.572 g/L by ultraviolet spectrophotometry (Additional file 4).

### Detection of rabbit anti-sheep ICOS serum titer through ELISA and western blotting identification

**Fig. 5:**
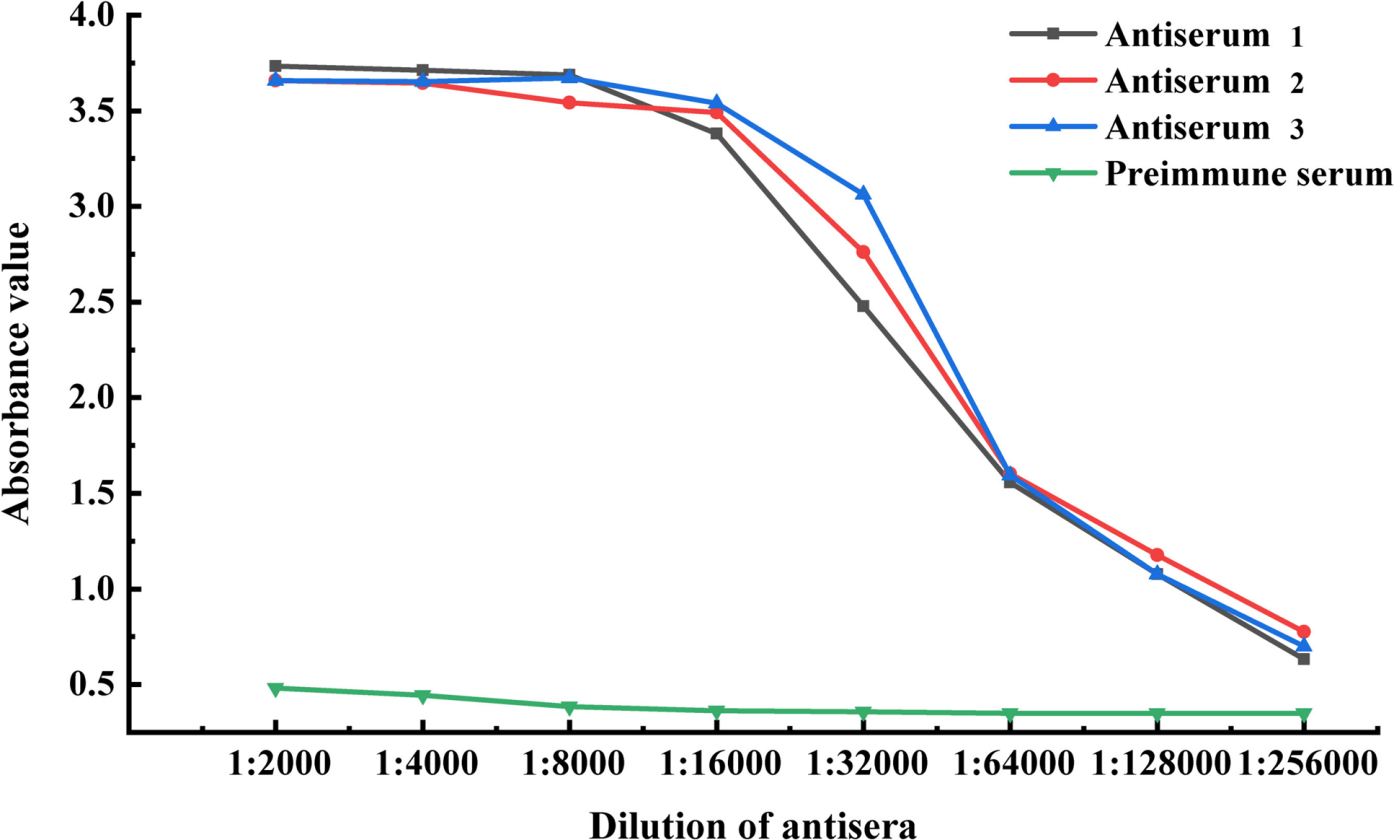
Determination of the antiserum titer by enzyme-linked immunosorbent assay (ELISA). The blue, red and black curves show the uptake values of antisera at different dilutions. The green curve shows the uptake values of the pre-immunization serum at the same dilution gradient (negative control)

The titer of the rabbit anti-sheep ICOS polyclonal antibody was determined using an indirect ELISA method. The antigen was coated at a concentration of 2.5 μg/ml, and the antisera was diluted with pre-immune negative sera at a ratio of 1:2000 to 1:128,000. The titer of polyclonal antibody was calculated to be not less than 1: 128,000 by D450 _nm (positive)_/D450 _nm (negative)_ ≥ 2.1 (Fig. 5).

The Western blotting results showed a distinct protein band at approximately 14.2 kDa (Fig. 4C), indicating that the rabbit anti-sheep ICOS antibody could specifically bind to the recombinant protein. There was a distinct protein band at approximately 23.8 kDa, and at approximately 23.8 kDa for the natural protein (Fig. 4C), indicating that the rabbit anti-sheep ICOS antibody could specifically bind to the natural protein (Additional file 4).

### Effect of *M. benedeni* infection on the expression of ICOS in the sheep small intestine

**Fig. 6:**
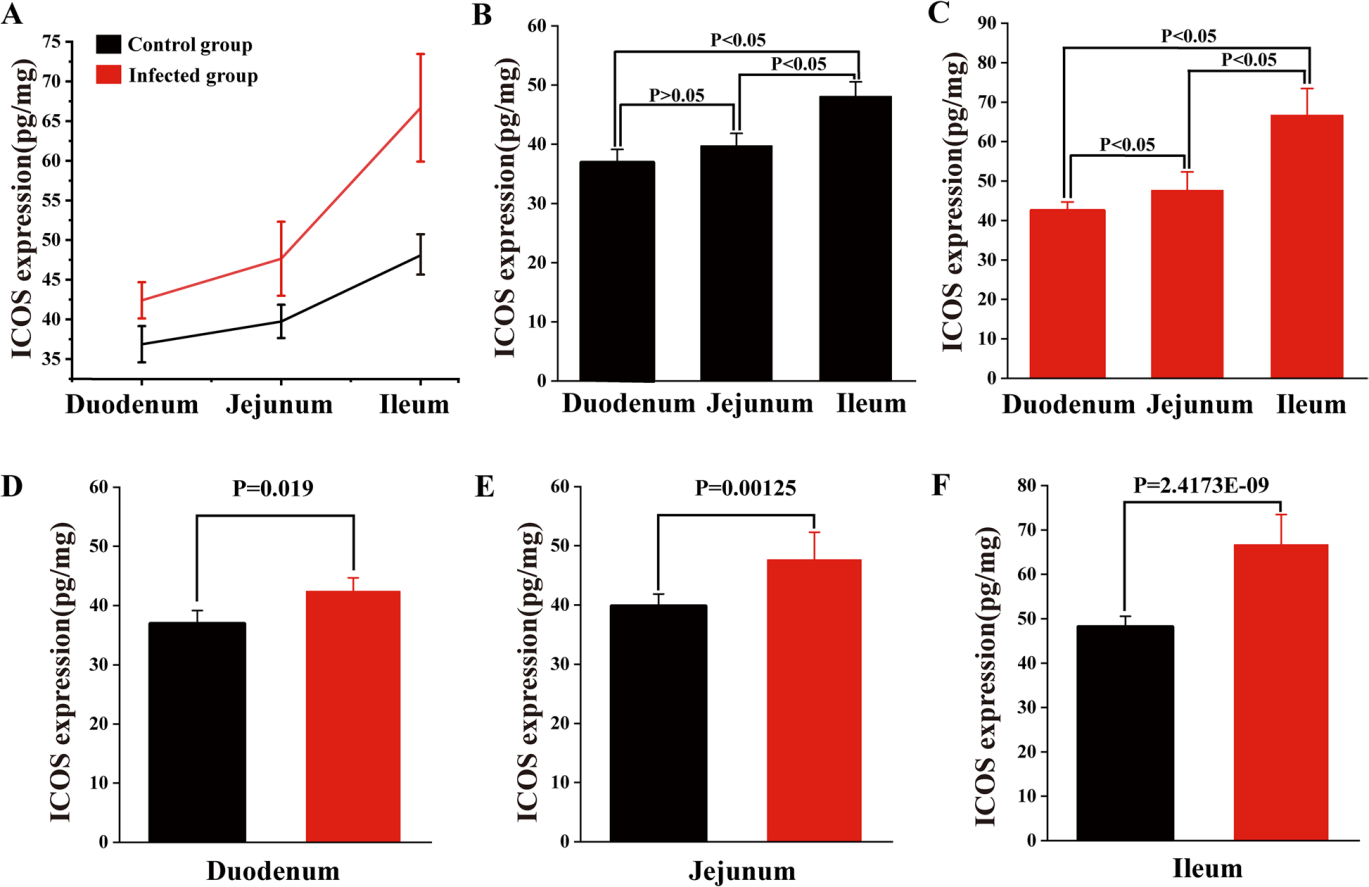
Expression level of ICOS in sheep small intestine. **A** ICOS expression in the sheep intestine from the duodenum to the ileum both in infected and control groups; **B** ICOS expression in the sheep intestine from the duodenum to the ileum both in control; **C** ICOS expression in the sheep intestine from the duodenum to the ileum both in infected; **D** ICOS expression in in duodenal; **E** ICOS expression in in jejunal; **F** ICOS expression in in Ileum. *P* < *0.05* means significant difference

The ELISA test results showed a gradual increase trend in ICOS expression in the sheep intestine from the duodenum to the ileum both in infected and control groups (Fig. 6A). There was no significant difference in the ICOS expression between the duodenum and jejunum in the control group (*P* > *0.05*), but there was a significant difference in the expression of the duodenum and ileum, as well as the jejunum and ileum (*P* < *0.05*) (Fig. 6B). In the infected group, the three intestine segments were significantly different from each other (*P* < *0.05*) (Fig. 6C). Infection with *M. benedeni* led to a significant increase in ICOS expression in all intestinal segments (Fig. 6D-F): the ileum showed the highest increase in expression level (*P* < *0.05*), followed by the jejunum (*P* < *0.05*) and duodenum (*P* < *0.05*).

### The impact of *M. benedeni* infection on the distribution characteristics of ICOS^+^ T cells and ICOS expression in the sheep small intestine

**Fig. 7:**
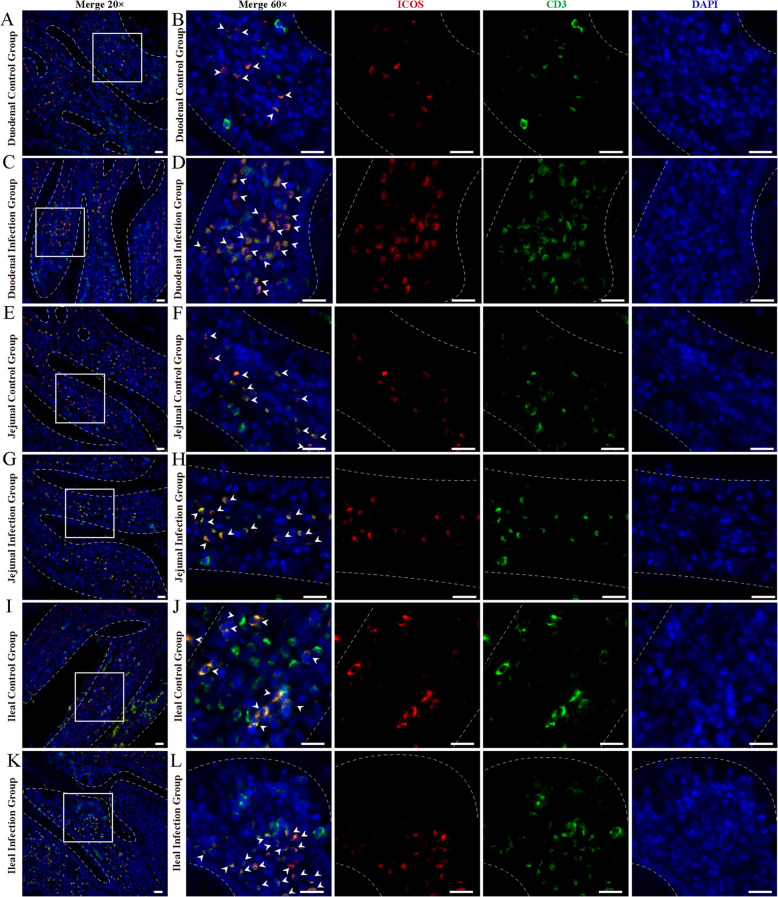
Immunofluorescence staining of ICOS^+^ T cells in the small intestine intraepithelial of control and infected sheep. **A**-**B** Duodenum of sheep in control group; **C**-**D** Duodenum of sheep in infected group; **E**–**F** Jejunum of sheep in control group; **G**-**H** Jejunum of sheep in infected group; **I**-**J** Ileum of sheep in control group; **K**-**L** Ileum of sheep in control group; CD3 (green), ICOS (red) and DAPI (blue). A-L Scale bars = 20 μm; white arrows show ICOS^+^ T cells

**Fig. 8:**
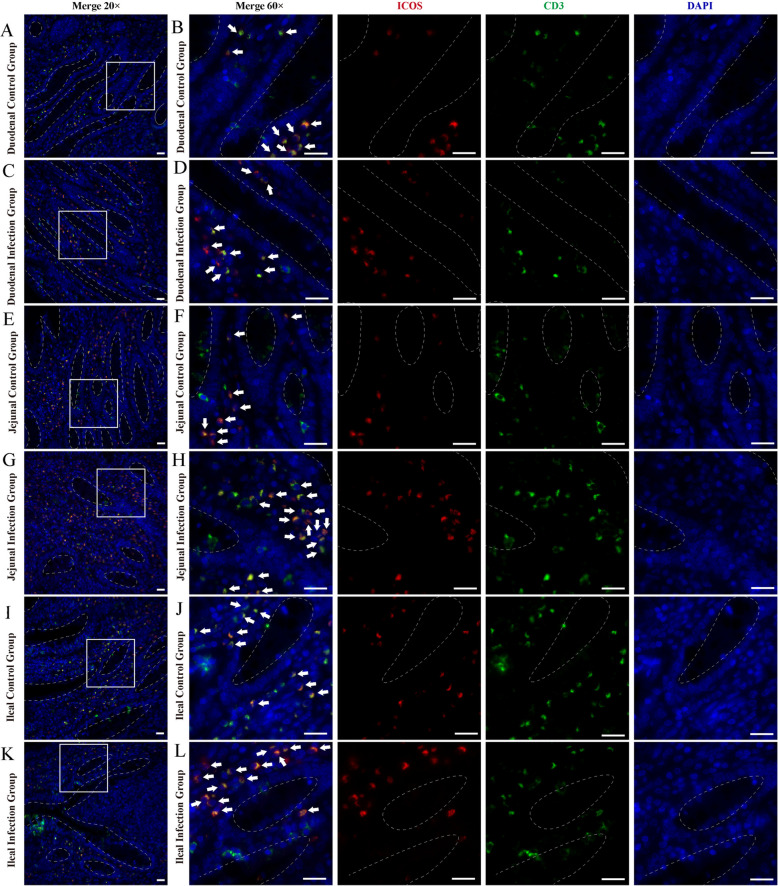
Immunofluorescence staining of ICOS^+^ T cells in the small intestine lamina propria of control and infected sheep. **A**-**B** Duodenum of sheep in control group; **C**-**D** Duodenum of sheep in infected group; **E**–**F** Jejunum of sheep in control group; **G**-**H** Jejunum of sheep in infected group; **I**-**J** Ileum of sheep in control group; **K**-**L** Ileum of sheep in control group; CD3 (green), ICOS (red) and DAPI (blue). A-L Scale bars = 20 μm; white arrows show the ICOS^+^ T cells

**Fig. 9:**
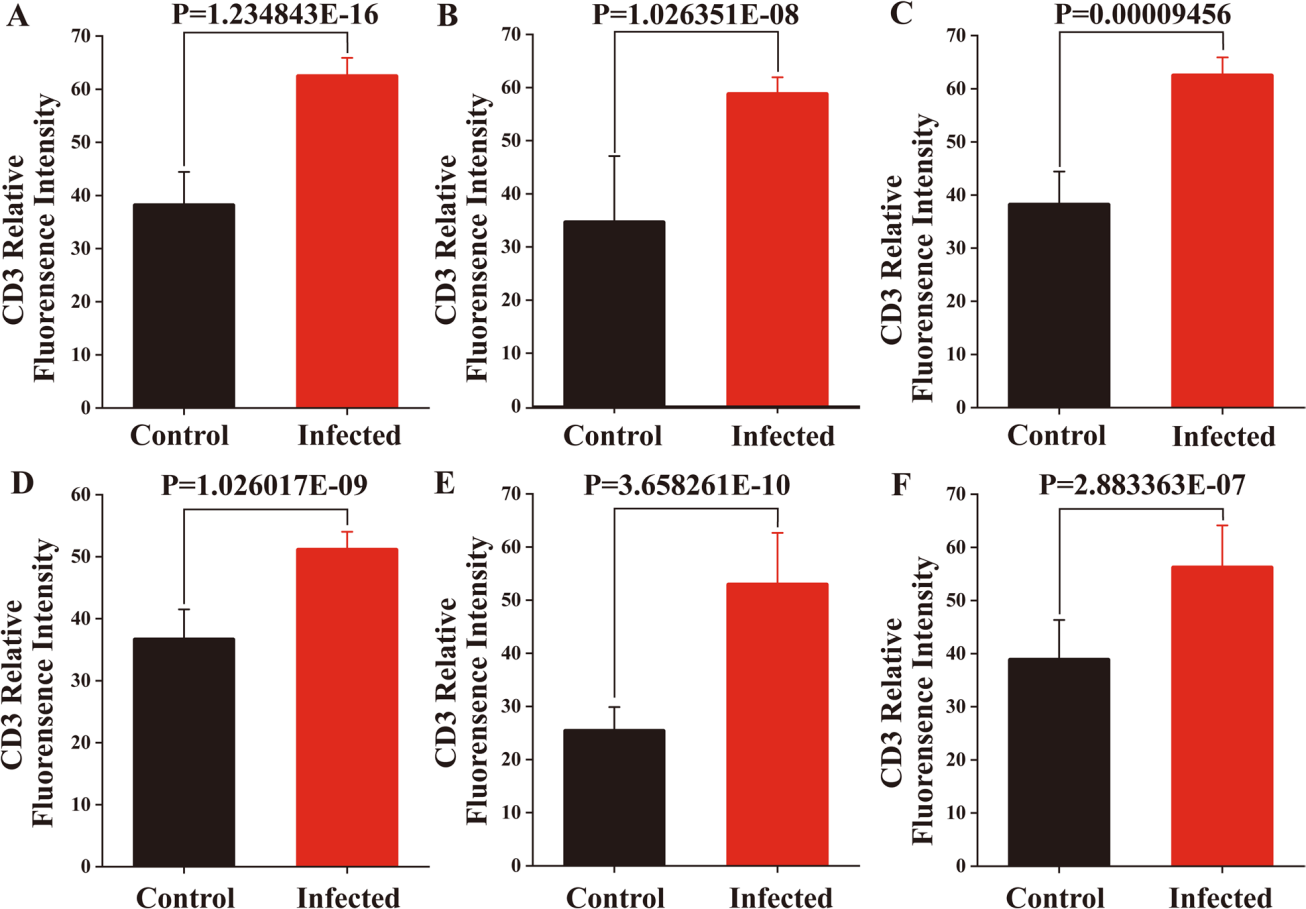
Immunofluorescence grey scale analysis results of CD3 in IEL and LPL in sheep small intestine. **A**-**D** The relative fluorescence intensities of CD3 in duodenal IEL and LPL; **B**-**E** The relative fluorescence intensities of CD3 in jejunal IEL and LPL; **C**-**F** The relative fluorescence intensities of CD3 in ileal IEL and LPL. IEL (Intestinal intraepithelial lymphocytes); LPL (lamina propria lymphocyte). *P* < *0.05* means significant difference

**Fig. 10:**
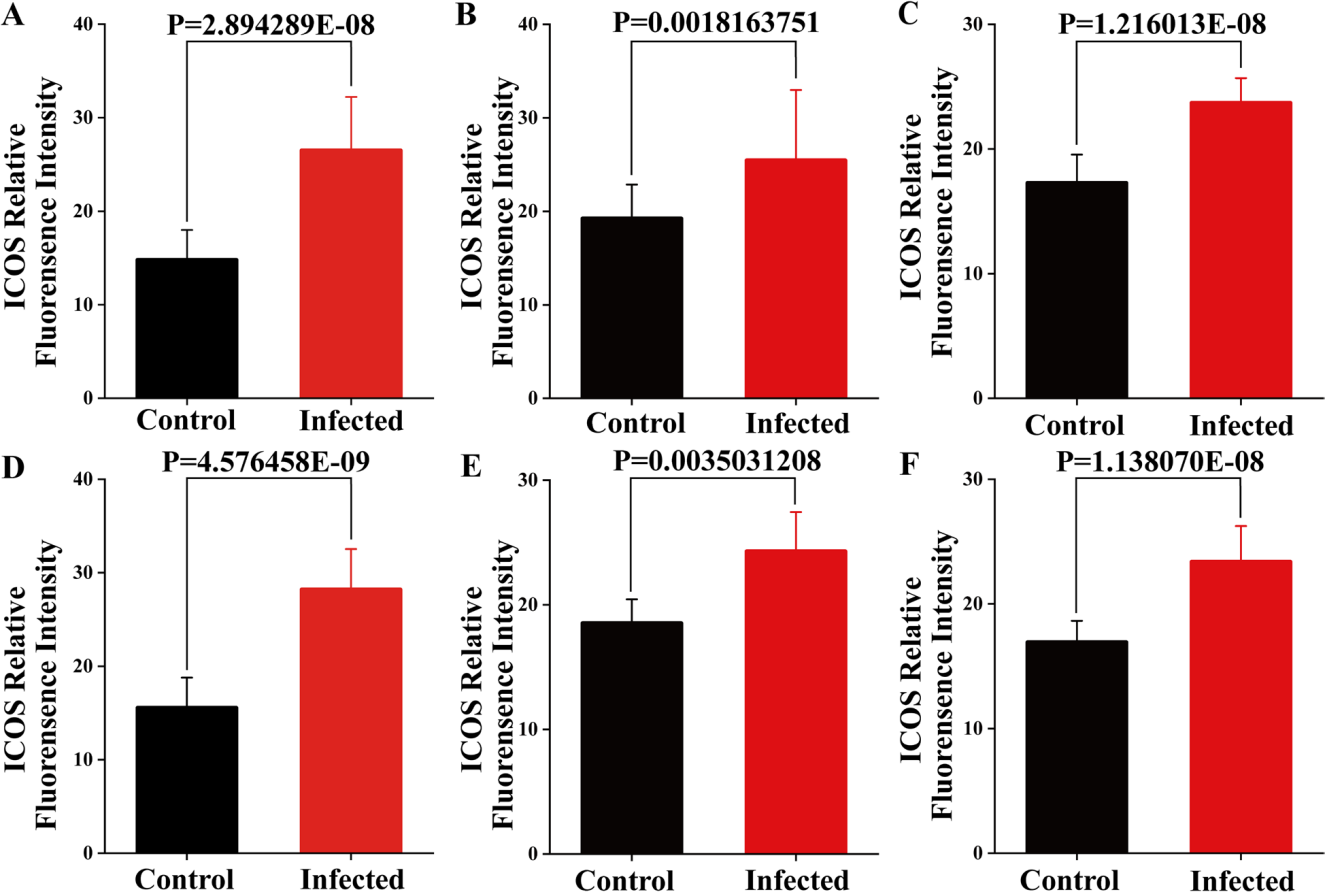
Immunofluorescence grey scale analysis results of ICOS in IEL and LPL in sheep small intestine. **A**-**D** The relative fluorescence intensities of ICOS in duodenal IEL and LPL; **B**-**E** The relative fluorescence intensities of ICOS in jejunal IEL and LPL; **C**-**F** The relative fluorescence intensities of ICOS in ileal IEL and LPL. IEL (Intestinal intraepithelial lymphocytes); LPL (lamina propria lymphocyte). *P* < *0.05* means significant difference

Immunofluorescence results showed that ICOS^+^ T cells were diffusely distributed within the epithelium of the duodenum, jejunum and ileum of sheep and around the intestinal glands in the lamina propria of the intestinal mucosa (Figs. 7 and 8). And there were no differences in the distribution sites, but the number increased between the infected and control groups. Analysis of the relative expression levels of the average fluorescence intensities of CD3 and ICOS using Image J reveals that infection with *M. benedeni* significantly elevates their expression levels (*P* < *0.05*). Through comparison, CD3^+^ cells exhibited the most remarkable change in the duodenal intestinal epithelium (*P* < *0.05*). In the lamina propria, CD3^+^ cells in the jejunum showed the most significant change (*P* < *0.05*). CD3^+^ cells were significantly increased in all intestinal segments of the infected group (*P* < *0.05*) (Fig. 9), consistent with the previous research results of our laboratory [33]. ICOS^+^ cells demonstrated the most pronounced change in the lamina propria of the duodenum (*P* < *0.05*). The most significant alteration within the intestinal epithelium was found in the duodenal intestinal epithelium. ICOS^+^ cells were significantly increased in all intestinal segments of the infected group (*P* < *0.05*) (Fig. 10). The above results indicated that ICOS^+^ T cells are widely distributed from the duodenum and jejunum to the intestinal epithelium and the lamina propria of the ileum. Moreover, after being infected with *M. benedeni*, the number of ICOS^+^ T cells in all intestinal segments significantly increases (*P* < *0.05*), with the most significant increase in the intestinal epithelium of the duodenum.

## Discussion

Predicting protein structure, function, and homology from amino acid sequences is a highly effective method for understanding protein properties. In this study, we analyzed the biological properties of sheep ICOS gene and protein using various prediction methods. The coding sequence (CDS) region of the sheep ICOS gene is 630 bp long, encoding 209 amino acids. The genetic distance between sheep and goats is the closest, up to 100%, and the farthest genetic distance is from protochickens, at 54%. The ICOS gene seems to be highly conserved in ruminants. The analysis results of the physicochemical properties of sheep ICOS protein showed that it has an isoelectric point of 6.11, an instability coefficient of 34.43, and a high number of hydrophilic amino acids than hydrophobic ones. Therefore, sheep ICOS protein is an acidic, stable, and hydrophilic protein that primarily exists in the cell membrane. The amino acid composition analysis showed that serine, tyrosine, and threonine were the most abundant, indicating that sheep ICOS is prone to phosphorylation modification. Phosphorylation and glycosylation analysis results showed that 18 sites could be protein kinase phosphorylation sites, and 2 asparagines could be protein kinase glycosylation sites. Therefore, it can be further speculated that phosphorylation is the main post-translational modification of this protein. Based on the above analysis, 123 amino acids in the extracellular region after intercepting the signal peptide were selected to construct the recombinant plasmid. The recombinant protein was successfully purified and polyclonal antibody with good specificity was prepared by immunizing rabbits.

Referring to the sheep tissue atlas included in NCBI (PMID 24904168), the sheep ICOS gene was mainly expressed in immune-related organs such as mesenteric lymph nodes, prescapular lymph nodes, Peyer’s patches and spleen. The proportion of irregularly curled regions in the secondary structure of ICOS was the highest, which predicted that there was a larger region of binding sites, indicating that ICOS could bind to a variety of proteins and thus perform a more complex immunologic function. Protein interaction network map analysis showed that sheep ICOS could have strong interactions with ICOLSG, CD40LG, CD4, BCL6, PIK3R1, PIK3R3, CTLA4, PIK3CA, PIK3CB, and CD40. Phosphatidylinositide 3-kinases (PI3Ks) are intracellular phosphatidylinositol kinases, a dimerized complex consisting of a catalytic subunit composed of PIK3CA and PIK3CB and a regulatory subunit composed of PIK3R1 and PIK3R3 [34]. The YXXM motif in the cytoplasmic tail of ICOS is the recognition site of the p85 subunit of PI3K, and ICOS binding to PI3K phosphorylates PIP2 to PIP3, thereby activating the AKT-A kinase to promote cell proliferation and survival [35]. Both ICOS and CTLA4 have the ability of bind to PI3K, mediating the PI3K-AKT signaling pathway [1, 36]. However, the immune functions mediated by ICOS and CTLA4 are different. ICOS up-regulates T-cell function, while CTLA4 down-regulates T-cell function [37]. Compared to CTLA4, the cytoplasmic domain of ICOS lacks Asn residues in the Tyr-Met-Phe-Met motifs. Therefore, ICOS cannot bind to growth-factor receptor-bound protein 2 (GRB2) and induce the production of IL-2, resulting in function and signaling differences [36, 38]. CD40 is expressed on antigen-presenting cells [39] (e.g., dendritic cells and B cells) and its receptor CD40LG is transiently expressed on the surface of activated CD4^+^ T cells [40]. Immunoglobulin class switching defect and impaired germinal center formation have been found in ICOS^−/−^ mice. In contrast, immunoglobulin isoform switching of ICOS^−/−^ mice could be restored under the stimulation of CD40 [41]. ICOS-ICOSL could promote the expression of CD40L on activated CD4^+^ T cells, thereby strengthening the CD40-CD40L interaction and providing a co-stimulatory signal for B cell activation. Therefore, ICOS-ICOSL and CD40-CD40L form a positive feedback loops, coordinating the terminal differentiation of B cells, including the formation of germinal centers, isotype switching, and the development of memory B cells [42]. Tfh cells are primarily distributed in lymphoid follicles and germinal centers, and plays a crucial role in germinal center formation, antibody class switching, B cell activation, and long-lived plasma cell maintenance [43]. ICOS is a key early signal for activation of BCL6, which can induce CXCR5 expression in Tfh cells [44]. CXCR5 promotes the chemotaxis of Tfh s to the lymphoid follicles, thereby promoting the formation of germinal centers [8]. In summary, ICOS has a complex regulatory process, suggesting that it plays a variety of functions in the immune system.

CD3 and ICOS co-localization could effectively label ICOS^+^ T cells. Immunofluorescence results showed that ICOS^+^ T cells were diffusely distributed in the intestinal epithelium and lamina propria of sheep duodenum, jejunum, and ileum, and there was no significant difference in the distribution location. CD3 and ICOS expression levels were significantly increased in all intestinal segments following *M. benedeni* infection (*P* < *0.05*), And the number of ICOS^+^ T cells in all intestinal segments significantly increases (*P* < *0.05*), with the most significant increase in the intestinal epithelium of the duodenum, indicating that ICOS^+^ T cells may play a crucial role in regulating the process of resistance to *M. benedeni* infection. The Th2 cell-mediated type II immune response plays a key role in protecting the host from parasitic helminth infection and in repairing tissue damage [45]. Studies have found that ICOS plays a dominant role in promoting the differentiation of Th2 cells and/or their effector functions through signal transduction mediated by IL-4R [46]. Experiments in ICOS^−/−^ mouse models have found that ICOS plays a key role in the differentiation and effector functions of both Th1 and Th2 cell types [29, 47]. ICOS expression is higher in Th2 cells than that in Th1 cells, but there is no valid evidence to determine whether ICOS selectively balances the transcription factors associated with Th1 and Th2 cells [30]. However, it is clear that during parasitic infection, ICOS can effectively promote parasite expulsion through the Th2 immune response [1]. The proportion of ICOS expression in CD4^+^ Foxp3^+^ regulatory T-cells (Treg cells) and CD4^+^ Foxp3^−^ effector T-cells (Teff cells) was significantly increased in the ICOS^−/−^ mice infected with *H.polygyrus* and *S.mansoni* parasites exhibited a significant increase, as well as a significant increase in IL-10, thereby promoting parasite control [31]. Studies have also demonstrated that ICOS can promote the proliferation and function of Foxp3^+^ Treg cells during various helminth infections [31]. Malaria is one of the most significant parasitic disease worldwide. Malaria uses ICOS^+^ T cells to promote the expression of the transcription factor T-bet and the secretion of the cytokine IFN-γ to promote its growth. Therefore, controlling the expression and signal transduction of ICOS can help change the parasite’s growth rate and lethality [48]. During Plasmodium chabaudi chabaudi AS infection, Tfh cells produce parasite-specific antibodies in lymphoid follicles of lymph nodes by providing co-stimulatory signaling to auxiliary B cells via ICOS. ICOS^−/−^ mice are significantly deficient in antibody production in the immune response, suggesting that ICOS signaling plays an important role in regulating antibody production [49].

From 2017 to 2024, our research team collected clinical specimens from sheep with suspected parasitic infections. Using a combination of clinical symptom evaluation, fecal egg examination, and gastrointestinal histopathological analysis, we identified six cases of single *M. benedeni* infection as valid samples for the infection group. Although these specimens were derived from naturally infected sheep in clinical settings, the sample size (*n* = 6) satisfied fundamental requirements for scientific investigation. Further accumulation of clinical infection cases will facilitate deeper exploration into the mechanisms by which *M. benedeni* impacts mucosal immunity in the sheep digestive tract.

In conclusion, ICOS^+^ T cells play a crucial role in anti-parasitic immunity, both by participating in the regulation of the development of immune responses by helper T cells and by regulating antibody production. Previous research results from our laboratory have shown that *M. benedeni* incontinentia infection does not alter the diffuse distribution characteristics of IgA^+^, IgG^+^, and IgM^+^ cells in the sheep small intestine. However, it significantly reduced their distribution density in the sheep small intestine [50], There is a potential regulatory relationship between ICOS^+^ T cells. Therefore, the analysis and experiment of sheep ICOS^+^ T cells can provide a basis for further research on the mechanism of action of ICOS in sheep anti-parasite regulation. Moreover, the rabbit anti-sheep ICOS polyclonal antibody prepared in this study has high specificity and can accurately locate the distribution of ICOS protein in sheep tissues. Therefore, it can be used to rapidly identify the expression of this protein under different pathological conditions.

## Conclusions

In this study, bioinformatics analysis showed that sheep ICOS protein is acidic, stable and hydrophilic, which mainly exists in cell membrane, and phosphorylation is the main way of dilution after translation of modified protein. The successfully expressed sheep recombinant ICOS protein which showed good immunogenicity and regenicity, and rabbit polyclonal antibody against sheep ICOS was successfully prepared by using the recombinant protein. The prepared polyclonal antibody has high specificity and can bind to natural protein. The expression of ICOS protein in ileum was significantly higher than that in jejunum and duodenum. ICOS^+^ T cells are diffusely distributed in the intestinal epithelium and around the intestinal glands in the lamina propria of the duodenum, jejunum, and ileum of sheep. However, *M. benedeni* infection did not change the spatial distribution of ICOS^+^ T in the small intestine, but the number of ICOS^+^ T cells in all intestinal segments significantly increases, with the most significant increase in the intestinal epithelium of the duodenum. It is suggested that *M. benedeni* infection can promote the expression of ICOS, which lays a foundation for further research on how ICOS regulates the T cell dependent immune response process to exert the immunity against parasitic infection. This study lay the foundation for further revealing the molecular mechanism of anti-parasite in the cellular immune process of sheep digestive tract.

## Supplementary Information

Supplementary Material 1.

Supplementary Material 2.

Supplementary Material 3.

Supplementary Material 4.

## Data Availability

The datasets generated and analyzed during the current study are available in the NCBI RefSeq repository (https://www.ncbi.nlm.nih.gov/refseq/) under accession numbers XM_004004846.4 (*ICOS* mRNA) and XP_004004895.2 (ICOS protein).
