# Supplementary material for: Moniezia benedeni infection promoting ICOS+ T cell proliferation in sheep (Ovis aries) small intestine
Source: BMC Vet Res. 2025 May 3;21:315. doi: 10.1186/s12917-025-04761-5 (PMC12048972; doi:10.1186/s12917-025-04761-5)
Supplement: Supplementary file 1 — Supplementary Material 1. [file 12917_2025_4761_MOESM1_ESM.pdf]

According to the coding region (CDS) region of ICOS nucleotide sequences of various species obtained from NCBI database.

>XM_004004846.4:52-681 PREDICTED: Ovis aries inducible T cell costimulator (ICOS), transcript variant X1, mRNA
ATGAAGTCAGACCTCTGGTATTTCTTTCTCTTCTGCACCCAAGTTGAAATTC
TAGCAGGAGAATTCAATG
ATTCTGCTGCATCTGAGATGTTCATATTTCACAATGGAGGTGTACAAATTTTA
TGCAAATACCCTGATAC
TGTTCGACAATTTAAAATGCAGTTGCTGAAAGGGGATAATGTACTCTGTGAT
CTCACTAAGATTAAGGGA
AGTGAAGACACGTTATCCACCAAGAATCTGAATGTCTGTAAATTTCAGTTAT
CCAATAATAGTGTCTCTT
TTTTTCTATATAATTTGGACAGTTCTTATGCCAGCTATTACATCTGCAAATTGT
CAATTTTTGATCCTCC
TCCTTTTCAAGTAGATATTCTAAGCAGAGAATATTTGAATATTTATGAATCAC
AGCTTTGTTGCCAGCTG
AAGTTCTGGTTACCCATAGGATGTGCAGCTTTTGTTATAGTCTGCGTTTTTG
GATGTGTCCTTATGTTTT
GGCTTACAAAAAAGAAGTATCCCACCAGCGTGCATGACCCTAACAGTGAAT
ACATGTTCATGGCAGCAGT
GAACACTGCTAAAAAGCCGGCACCCACAGATGTGACCCGTAATTTGGAAC
TCCCTGGCACCCAGGCATGA
>XM_006496138.3:845-1447 PREDICTED: Mus musculus inducible T cell co-stimulator (Icos), transcript variant X1, mRNA
ATGAAGCCGTACTTCTGCCGTGTCTTTGTCTTCTGCTTCCTAATCAGACTTT
TAACAGGAGAAATCAATG
GCTCGGCCGATCATAGGATGTTTTCATTTCACAATGGAGGTGTACAGATTTC
TTGTAAATACCCTGAGAC
TGTCCAGCAGTTAAAAATGCGATTGTTCAGAGAGAGAGAAGTCCTCTGCG
AACTCACCAAGACCAAGGGA
AGCGGAAATGCGGTGTCCATCAAGAATCCAATGCTCTGTCTATATCATCTGT
CAAACAACAGCGTCTCTT
TTTTCCTAAACAACCCAGACAGCTCCCAGGGAAGCTATTACTTCTGCAGCC
TGTCCATTTTTGACCCACC
TCCTTTTCAAGAAAGGAACCTTAGTGGAGGATATTTGCATATTTATGAATCC
CAGCTCTGCTGCCAGCTG
AAGCTCTGGCTACCCGTAGGGTGTGCAGCTTTCGTTGTGGTACTCCTTTTTG
GATGCATACTTATCATCT
GGTTTTCAAAAAAGAAATACGGATCCAGTGTGCATGACCCTAATAGTGAAT
ACATGTTCATGGCGGCAGT

CAACACAAACAAAAAGTCTAGACTTGCAGGTGTGACCTCATAA
>XM_008259011.3:926-1594 PREDICTED: Oryctolagus cuniculus inducible T cell costimulator (ICOS), mRNA
ATGAATTCGTTGTCAGCTCTGAAGACTGAAGGAGGAGGGTGCGTGCTTCT
GGCAAACATGAAGTCAGACC
TTTGGTATTTCTTTCTCTTCTGCTTCCAGGTTGAAGTTCTAACGGGAGAAAT
CAATGACTCTGCCAAGTA
TGAGATGTTTACATTTCACAATGGAGGTGTTCAAATTGTATGCAAATACCCC
GAGATTGTCCAGCAATTC
AAAATGCAGCTACTGAAAGGGGAACAAGTCCTCTGTGACCTCAGGCAGAC
AAAGGAAAGTGGAAACACAG
TGTCCATTAAGGCTCTGAAATTCTGTCAATATCAATTATTCAACAACAGCGT
CTCTTTTTTTCTGTATAA
TTTGGACAGTTCTCATGCCAGCTATTACTTCTGCAAACTATCAACTTTTGAT
CCTCCTCCTTTTCAAGTA
GAGATTCTTAGAGGAGAATATTTGCATATTTATGAATCACAGCTCTGTTGCC
AGCTGAAGTTCTGGTTAC
CCATAGGATGCGCAGCTTTTGCAGTAGTCTACATTTTTGGATGTGTATTTATT
TTCTGGCTTACAAAAAA
GAAGTATCAGTCCAGTGTGCATGACCCGAATAGTGAGTACATGTTCATGGC
AGCAGTGAACACAGCTAAA
AAACCTACACCGCCAGAACTCTCTGGCACCCGGGCGTGA
>NM_001100288.2:19-678 Gallus gallus inducible T-cell costimulator (ICOS), mRNA
ATGCCTCTATGTCAGGCAAGCATGAAGACAGTTGCAGTAACTTTCTGTCTC
CTCTGCTTTCAGTTTGAAG
CCTTGTGTGGAGTGGATACTTGCTCATCAAGATTGTGCAAAAATATAGATAA
GCTTCAGGTCTCGGACCC
CCAGGGGATAGTAGAATTTGAAAATGGAAACTTCAAGTTAATATTTCAAAA
CCCCAAAAATGTGAACGAG
TTCAGCATGACCCTCCTCAAAGGGCGAGAAAGGAAGGCAATCTGTGCACT
CCATATGAATAATAAGAAAG
CTGTCCCAGAGAGTAATGTCACCTACTGCCAGGCAGAGCATTCAGATACCA
GCACCACCTTCATTCTCAC
AAATCTGGACAGAAAGCACATCGACACTTATACCTGCTGCCTGGAAAGTTT
ATTACCCCCCCCTTACATA
CATTGCCACTTGAAAGAAACCTATTTGTACATCCAAGATAAGGAAGACTGC
TCTTCACAAGGAATCATGT
CATGGATAATTATTGGCCTGATTGCATTTGCCCTGATTTTCTGTGTCTGCTTT
GTAGTAGCCTGTCACTT
AAGGAATAAGAATCAGCAGTGTGAATCCAACTCCCATGAGTACAACAGTG
AATACATGCCCATGGCAGCA
GTGAATGCAGCTAAAAAACCAAGAATCTGA
>XM_005676405.3:49-678 PREDICTED: Capra hircus inducible T-cell costimulator (ICOS), mRNA

ATGAAGTCAGACCTCTGGTATTTCTTTCTCTTCTGCACCCAAGTTGAAATTC
TAGCAGGAGAATTCAATG
ATTCTGCTGCATCTGAGATGTTCATATTTCACAATGGAGGTGTACAAATTTTA
TGCAAATACCCTGATAC
TGTTCGACAATTTAAAATGCAGTTGCTGAAAGGGGATAATGTACTCTGTGAT
CTCACTAAGATTAAGGGA
AGTGAAGACACGGTGTCCACCAAGAATCTGAATGTCTGTAAATTTCAGTTA
TCCAATAATAGTGTCTCTT
TTTTTCTATATAATTTGGACAGTTCTTATGCCAGCTATTACATCTGCAAATTGT
CAATTTTTGATCCTCC
TCCTTTTCAAGTAGATATTCTAAGCAGAGAATATTTGAATATTTATGAATCAC
AGCTTTGTTGCCAGCTG
AAGTTCTGGTTACCCATAGGATGTGCAGCTTTTGTTATAGTCTGCGTTTTTG
GATGTGTCCTTATGTTTT
GGCTTACAAAAAAGAAGTATCCCACCAGCGTGCATGACCCTAACAGTGAAT
ACATGTTCATGGCAGCAGT
GAACACTGCTAAAAAGCCGGCACCCACAGATGTGACCCGTAATTTGGAAC
TCCCTTGCACCCAGGCATGA
>XM_015239991.2:105-734 PREDICTED: Vicugna pacos inducible T cell costimulator (ICOS), mRNA
ATGAAGTCAGACTTCTGGTATTTCTTTCTCCTCTGCTTCCAAGTTGAAGTTC
TCACAGGAGAATTCAATG
ATTCTGCCAAGTCTGAGATGTTTATATTTCACAATGGAGGTGTACAGATCTT
ATGCAAATACCCTGATAT
TGTCCGACAATTTAAAATGCAGTTGCTGAAAGGGAAGAACACGCTCTGTG
AGCTCAGTAAGACACAGGAA
AGCGGAAACACGGTCTCCATTAAGAATCCGAACTTCTGTCAATTTGAATTAT
CCAATAACAGTGTCTCTT
TTTTCCTGTATAACTTGGATAGTTCTCATGCCAGCTATTACATCTGCAAACTA
TCAATTTTTGATCCTCC
TCCTTTTCATATAGATATACTAAGCAGAGAATATTTGAATATTTATGAATCAC
AGCTTTGTTGCCAGCTG
AAGTTCTGGTTACCCATAGGATGTGCAGCTTTTGTTGTAGTCTACATTTTTG
GATTCGTCCTGACATGTT
GGCTTACAAAAAAGAAGTACCGCTCCAGCGTGCACGACCCTAACAGTGAG
TACATGTTCATGGCTGCAGT
GAACACCGCGAAGAAGCCTAGACTCGCAGATGTGACCCCTAATTTGGAAC
TCTCTGGCACCCAGGCATGA
>XM_010352637.2:85-684 PREDICTED: Rhinopithecus roxellana inducible T cell costimulator (ICOS), mRNA
ATGAAGTCAGGCCTCTGGTATTTCTTTCTCTTCTGCTTGCACATGAAAGTTG
TAACAGGAGAAATCAATG
GTTCTGCCAATTATGAGATGTTTATATTTCACAACGGAGGTGTACAAATTTTA
TGCAAATATCCTGACAT

TGTCCAGCAATTTAAAATGCAGTTGCTGAAAGGGGGGCAAATACTCTGCGA
TCTCACTAAGACAAAAGGA
AGTGGAAACACAGTGTCCATTAAGAGTCTGAAATTCTGCCATTCTCAGTTA
TCCAACAACAGTGTCTCTT
TTTTTCTATATAACTTGGACCATTCTCATGCCAACTATTACTTCTGCAACCTA
TCAATTTTTGATCCTCC
TCCTTTTAAAGTAACTCTTACGGGAGGATATTTGCATATTTATGAATCACAAC
TTTGTTGCCAACTGAAG
TTCTGGTTACCCATAGGATGTGCAACCTTTGTTGTTGTCTGCATTTTTGCGT
GCATACTTATTTGTTGGC
TTACAAAAAAGAAGTATTCATCCAGCGTGCACGACCCTAACGGTGAATACA
TGTTCATGAGAGCAGTGAA
CACAGCCAAAAAATCTAGACTCACAGATGTGACCGTATAA
>XM_010991757.2:105-734 PREDICTED: Camelus dromedarius inducible T cell costimulator (ICOS), mRNA
ATGAAGTCAGACTTCTGGTATTTCTTTCTTCTCTGCTTCCAAGTTGAAGTTC
TTACAGGAGAATTCAATG
ATTCTGCCAAGTCTGAGATGTTTATATTTCACAATGGAGGTGTACAGATTTT
ATGCAAATACCCTGATAT
TGTCCGACAATTTAAAATGCAGTTGCTGAAAGGGAAGAACACGCTCTGTG
AGCTCAGTAAGACACAGGAA
AGCGGAAACACGGTCTCCATTAAGAATCCGAACTTCTGTCAATTTGAATTAT
CCAATAACAGTGTCTCTT
TTTTCCTGTATAACTTGGATAGTTCTCATGCCAGCTATTACATCTGCAAACTA
TCAATTTTTGATCCTCC
TCCTTTTCATATAGATATTCTAAGCAGAGAATATTTGAATATTTACGAATCAC
AGCTTTGTTGCCAGCTG
AAGTTCTGGTTACCCATAGGATGTGCAGCTTTTGTTGTAGTCTACATTTTTG
GATTCGTCCTTACATGTT
GGCTTACAAAAAAGAAGTACCGCTCCAGCGTGCACGACCCTAACAGTGAG
TACATGTTCATGGCTGCAGT
GAACACCGCGAAGAAGCCTAGACTCACAGATGTGACCCGTAATATAGAACT
CTCTGGCACCCAGGCATGA
>XM_053905749.1:85-828 PREDICTED: Panthera pardus inducible T cell costimulator (ICOS), transcript variant X2, mRNA
ATGAAGTTAGACCTCTGCTATTTCCTTCTCTTCTGCTTTCAAGTTGAAGTCC
TAACAGGAGAAATCAATG
ATTCTGCCAAGTCCGAGATGTTTACCTTTCACGATGGAGGCATACAAATTTC
ATGCAAATTCAGTGAGAT
AGCCTTGCAATTTAAAATGAAGTTGCTAAAAGGGACGGAAGTACTCTGTGA
TCTCACTAAGACAAAAGAA
AGTGGTAACACGGTGTCCATTAAGAATCTGAAATTCTGTCAAACTCAGTTAT
TCAATGACAGTGTCTCCT
TTTTCCTGTATAATTTGGACAATTCTCATGCCAGCTATTACACCTGCGAACTG

TCAATTTTTGATCCTCC
TCCTTTTCAGAAGAAGAATATTAGCAGAGAATATTTGAATATTTATGAATCA
CAGATTTGCTGCCAGCTG
AAGTTCTGGTTACCCATAGGGTGTTCAGCTTTTGTTGTAGTCTACATTTTTG
GATGCGTATTTCTTTGTT
GGCTTACAAAAAAGAAGTATCGATCCAGTGGGCATGACCCTAACAGTGAAT
ATATGTTTATGGCAGCAGT
GAACACAGCCAAGAAACCTGGACTCACAGGCATGAACCACGTGGGCCAG
GTCCCCTCTGACTTGAAGCGC
AAGATTCCCACGTCCCCTGGACCACAGAGAGTCAGACTTGATTTGAGTACA
TGCATCTTCTGCTGGTGTT
CTGTTCAATCTGGACTAGTGACTATCGGTCAACAGGGGTTTTAA
>NM_001044546.3:36-665 Sus scrofa inducible T cell costimulator (ICOS), mRNA
ATGAAGTCAGATCTCTGGTATTTCTTTCTCTTCTGCTTCCAAGTTGAAGTTC
TAATGGGAGAAGTCAATG
ACTCTGCCAAGTCTGAGATGTTTATGTTTCATGATGGAGGTCTACAGATTGT
ATGCAAATATCCTGATAC
TGTCCGACAATTTAAGATGCAGTTGCTGAAAGGGAAGAATATACTCTGTGA
CCTTACGAAGACAAAGGGA
AGTGGAAACACGGTGTCCATTACCAACGTGAATTTATGCCAGTTTCAGTTAT
CCAATAACAGTGTCTCTT
TTTTTCTGTATAACTTGGACAGTTCTTATGCCAGCTATTACATCTGTGAATTA
TCAATTTTTGATCCTCC
ACCTTTTCAAGTAGATATTCTAAGCAAAGAATATTTGAATATTTATGAATCAC
AGCTTTGTTGCCAACTG
AAGTTCTGGTTACTTCTAGGATGTGCAGCCTTTGTTGTAGTCTACATTATTG
GATGTGTTCTTACATGTT
GGCTTACAAAAAAGAAATATCGCCCCAGCGTGCATGACCCTAATAGTGAAT
ACATGTTCATGGCAGCAGT
GAACACCACTAAAAAGGCTGGACCAACAGATGTGACCCGCAATTTGGTAC
GCTCTGGCACACGGGCATGA
>NM_001034275.1:7-636 Bos taurus inducible T cell costimulator (ICOS), mRNA
ATGAAGTCAGACCTCCGGTATTTCTTTCTCTTCTGCATCCAAGTTGAAATTC
TAGCAGGAGAATTCAATG
ATTCTGCTGCATCTGAGATGTTCATATTTCACAATGGAGGTGTACAAATTTTA
TGCAAATACCCTGATAC
TGTTCGACAATTTAAAATGCAGTTGCTGAAAGGGGATAATGTACTCTGTGAT
CTCACTAAGACTAAGGAA
AATGAAGACACAGTATCCATCAGGAATCTGAATGTCTGTAAATTTCAGTTAT
CCAATAATAGTGTCTCTT
TTTTTCTATATAATTTGGACAGTTCTTATGCCAGCTATTACATCTGCAAACTG
TCAATTTTTGATCCTCC
TCCTTTTCAAGTAGATATTCTAAGCAGAGAATATTTGAATATTTATGAATCAG
AGCTTTGTTGCCAGCTG

AAGTTCTGGTTACCCATAGGATGTGCAGCTTTTGTTACAGTCTGCGTTTTTG
GATGTGTCCTTATGTATT
GGCTTACAAAAAAGAAGTATCCCACCAGCGTGCATGACCCTAACAGTGAAT
ATATGTTCATGGCAGCGGT
GAACACTGCTAAAAAGCCTGCACCCACAGATGTGACCCGTAATTTGGAACT
CCCTGGCACCCAGGCATGA
>XM_001497868.6:73-699 PREDICTED: Equus caballus inducible T cell costimulator (ICOS), transcript variant X2, mRNA
ATGAAGTCAAACCTCTGCTATTTCTTTCTCTTCTGCTTCCAAGTTGAAGCTC
TCACAGGGAAAATTGGTG
ATTCTGCCAAGTCGGACATGTTTATAGCTCATGACGGGGGTGTCCAAATTTT
ATGCAAATATCCTGAGAC
CGTCCGGCAGTTTAAAATGCAGTTGCTGAAGGGGATGCAACCACTTTGCAA
CCTCACGAGGATGGAGGAG
AGTGGAAACACGGTGTCCGTCAAGAACCTGCAGCTCTGCCAGTCTCAGCT
GTCCAACAGCAGCGTCTCTT
TCTTCCTGTATAACCTGGACCGTTCTCATGCCAGCTACTACTCCTGCAAACT
CCTCATTTTTGATCCTCC
TCCTTTTCAAGAGATTGTTAGCACAGAATATTTGCATATTTATGAATCACAGC
TCTGTTGCCAGCTGAAG
CTCTGGTTGCCCATAGGATGTGCGGCTTTTCTGGTGGTCTACATTTTTGCCT
GTGTCCTTATATGTTGCC
TTGTAAAAAAGAAGCCTCGCCCCAGTGTGCACGACCCTAATAGTGAATACA
TGTTCATGGCTGCGGTGAA
CACGGCTAAGAAGCCTAGACTCGCAGATGTGAGCCGTCATTTGGAACTCTC
TGGCACCCAGGCGTGA
>NM_001002972.2:16-642 Canis lupus familiaris inducible T cell costimulator (ICOS), mRNA
ATGAAGTCAGACCTCTGGTATTTCCTTCTCTTCTGCTTTCAAGTTGAAGCCC
TAACAGGAGAAATCAATG
ATTCTACCAAGTCTGAGATGTTTACATTTCACGATGGAGGTGTACAAATTTT
ATGCAAATTCAATGCGAT
TGTCTCGCAATATAAAATGGAGTTGCTGAAAGGGACAGAAGTACTCTGTGA
TCTCACTACGACAAAGGAA
AATGGAAACACAGTGTCCAAGAATCCGAAATTCTGTCAATCTCAGTCATCC
AGTGATGGTGTCTCCTTTT
TTCTGTATAACTTGGACAGTTCTCATGCCAGCTACTATGCCTGCCAACTGTC
AATTTTTGATCCTCCTCC
TTTTCAGAGAAAAAATATTAGCAGAGAATATTTGAATGTTTATGAATCACAG
ACTTGCTGCCAACTGAAG
TTCTGGTTACCCATAGGATGTGCAGCTTTTGTTGTAGTCTATATTTTTGGATG
CATATTTCTTTGTTGGC
TTACAAAAAAGAAATATCGATCCAGTGTGCATGACCCTAACAGTGAATACA
TGTTCATGGCAGCAGTGAA

CACAGCCAAAAAACCTGGACTCACAGGTGTGACTCATAATTTGGAACTCTG
TGGCACCCAGGCATGA
>NM_012092.4:53-652 Homo sapiens inducible T cell costimulator (ICOS), mRNA
ATGAAGTCAGGCCTCTGGTATTTCTTTCTCTTCTGCTTGCGCATTAAAGTTT
TAACAGGAGAAATCAATG
GTTCTGCCAATTATGAGATGTTTATATTTCACAACGGAGGTGTACAAATTTTA
TGCAAATATCCTGACAT
TGTCCAGCAATTTAAAATGCAGTTGCTGAAAGGGGGGCAAATACTCTGCGA
TCTCACTAAGACAAAAGGA
AGTGGAAACACAGTGTCCATTAAGAGTCTGAAATTCTGCCATTCTCAGTTA
TCCAACAACAGTGTCTCTT
TTTTTCTATACAACTTGGACCATTCTCATGCCAACTATTACTTCTGCAACCTA
TCAATTTTTGATCCTCC
TCCTTTTAAAGTAACTCTTACAGGAGGATATTTGCATATTTATGAATCACAAC
TTTGTTGCCAGCTGAAG
TTCTGGTTACCCATAGGATGTGCAGCCTTTGTTGTAGTCTGCATTTTGGGAT
GCATACTTATTTGTTGGC
TTACAAAAAAGAAGTATTCATCCAGTGTGCACGACCCTAACGGTGAATACA
TGTTCATGAGAGCAGTGAA
CACAGCCAAAAAATCTAGACTCACAGATGTGACCCTATAA
