# Supplementary material for: Moniezia benedeni infection promoting ICOS+ T cell proliferation in sheep (Ovis aries) small intestine
Source: BMC Vet Res. 2025 May 3;21:315. doi: 10.1186/s12917-025-04761-5 (PMC12048972; doi:10.1186/s12917-025-04761-5)
Supplement: Supplementary file 2 — Supplementary Material 2. [file 12917_2025_4761_MOESM2_ESM.pdf]

Bioinformatics Analysis Software and Web Sites

Software and Web Sites	Website address	Object of analysis/forecasting
ProtParam	https://web.expasy.org/protparam/	Protein Physical and Chemical Properties
ProtScale	https://web.expasy.org/protscale/	Protein hydrophilicity/hydrophobicity
TMHMM2.0	https:// servi ces. healt htech. dtu. dk/ servi ce. php? TMHMM-2.0	Transmembrane Structure
SignalP-6.0	https://services.healthtech.dtu.dk/services/SignalP-6.0/	Signal peptide
Cell-PLoc 2.0	http://www.csbio.sjtu.edu.cn/bioinf/Cell-PLoc-2/	Subcellular localization
BIOINF	http://bioinf.cs.ucl.ac.uk/	Secondary structure
Swiss-Model	https://swissmodel.expasy.org/	Tertiary structure
NetPhos2.0	https://services.healthtech.dtu.dk/service.php?NetPhos-3.1	Phosphorylation sites
NETOGlyc3.1	https://services.healthtech.dtu.dk/service.php?NetNGlyc-1.0	Glycosylation sites
STRING	https://cn.string-db.org/	Protein interactions
