# Supplementary material for: Moniezia benedeni infection promoting ICOS+ T cell proliferation in sheep (Ovis aries) small intestine
Source: BMC Vet Res. 2025 May 3;21:315. doi: 10.1186/s12917-025-04761-5 (PMC12048972; doi:10.1186/s12917-025-04761-5)
Supplement: Supplementary file 3 — Supplementary Material 3. [file 12917_2025_4761_MOESM3_ESM.pdf]

Exhibit 2: ICOS gene synthesis and recombinant plasmid construction process

PREDICTED: Ovis aries inducible T cell costimulator (ICOS), transcript variant X1, mRNA

NCBI Reference Sequence: XM_004004846.4

ICOS base sequence (CDs) 630 bases

atgaagtcagacctctggtatttctttctcttctgcacccaagttgaaattctagcaggagaattcaatgattctgctgc
atctgagatgttcatatttcacaatggaggtgtacaaattttatgcaaataccctgatactgttcgacaatttaaaatgc
agttgctgaaaggggataatgtactctgtgatctcactaagattaagggaagtgaagacacgttatccaccaagaatctg
aatgtctgtaaatttcagttatccaataatagtgtctcttttttctatataatttggacagttcttatgccagctatta
catctgcaaattgtcaatttttgatcctcctccttttcaagtagatattctaagcagagaatatttgaatatttatgaat
cacagctttgttgccagctgaagttctggttacccataggatgtgcagcttttgttatagtctgcgtttttggatgtgtc
cttatgttttggcttacaaaaaagaagtatcccaccagcgtgcatgaccctaacagtgaatacatgttcatggcagcagt
gaacactgctaaaaagccggcacccacagatgtgacccgtaatttggaactccctggcacccaggcatga

Amino acid sequence of ICOS (CDs) 209 amino acids

MKSDLWYFFLFCTQVEILAGEFNDSAASEMFIFHNGGVQILCKYPDTVRQFK
MQLLKGDNVLCDLTKIKGSEDTLSTKNLNVCKFQLSNNSVSFFLYNLDSSYAS
YYICKLSIFDPPPFQVDILSREYLNIYESQLCCQLKFWLPIGCAAFVIVCVFGCV
LMFWLTKKKYPTSVHDPNSEYMFMAAVNTAKKPAPTDVTRNLELPGTQA.

Transmembrane region prediction analysis, amino acids 1-142 for the extramembrane fraction

MKSDLWYFFLFCTQVEILAGEFNDSAASEMFIFHNGGVQILCKYPDTVRQFK
MQLLKGDNVLCDLTKIKGSEDTLSTKNLNVCKFQLSNNSVSFFLYNLDSSYAS
YYICKLSIFDPPPFQVDILSREYLNIYESQLCCQLKF

Signal peptide prediction analysis, amino acids 1-19 are the signal peptide portion, truncated signal peptide, 123 amino acids remaining

GEFNDSAASEMFIFHNGGVQILCKYPDTVRQFKMQLLKGDNVLCDLTKIKGS
EDTLSTKNLNVCKFQLSNNSVSFFLYNLDSSYASYYICKLSIFDPPPFQVDILSR
EYLNIYESQLCCQLKF

The starting amino acid M was added separately, totaling 125 amino acids and 14.2 kDa, with the following results:

MGEFNDSAASEMFIFHNGGVQILCKYPDTVRQFKMQLLKGDNVLCDLTKIKG
SEDTLSTKNLNVCKFQLSNNSVSFFLYNLDSSYASYYICKLSIFDPPPFQVDILS
REYLNIYESQLCCQLKF

The start codon atg, and the stop codon tga, totaling 375 bases, were added separately, and the results were as follows:

atgggagaattcaatgattctgctgcatctgagatgttcatatttcacaatggaggtgtacaaattttatgcaaataccc
tgatactgttcgacaatttaaaatgcagttgctgaaaggggataatgtactctgtgatctcactaagattaagggaagtg
aagacacgttatccaccaagaatctgaatgtctgtaaatttcagttatccaataatagtgtctctttttttctatataat
ttggacagttcttatgccagctattacatctgcaaattgtcaatttttgatcctcctccttttcaagtagatattctaag
cagagaatatttgaatatttatgaatcacagctttgttgccagctgaagttctga

A+T = 65.6， C+G = 34.4， 14.2kDa.
