# Supplementary material for: Moniezia benedeni infection promoting ICOS+ T cell proliferation in sheep (Ovis aries) small intestine
Source: BMC Vet Res. 2025 May 3;21:315. doi: 10.1186/s12917-025-04761-5 (PMC12048972; doi:10.1186/s12917-025-04761-5)
Supplement: Supplementary file 4 — Supplementary Material 4. [file 12917_2025_4761_MOESM4_ESM.pdf]

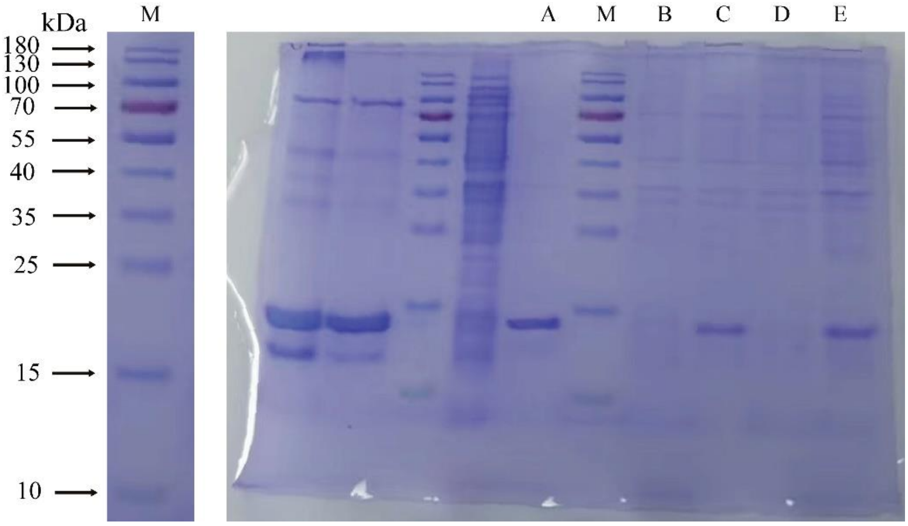

Figure 4 (A, B) Original Figure (A). Purified recombinant protein; (B). Pre-induction product of recombinant bacteria; (C). Post-induction product of recombinant bacteria; (D). Supernatant of induced product; (E). Precipitation of induced product; (M). Protein molecular weight marker.

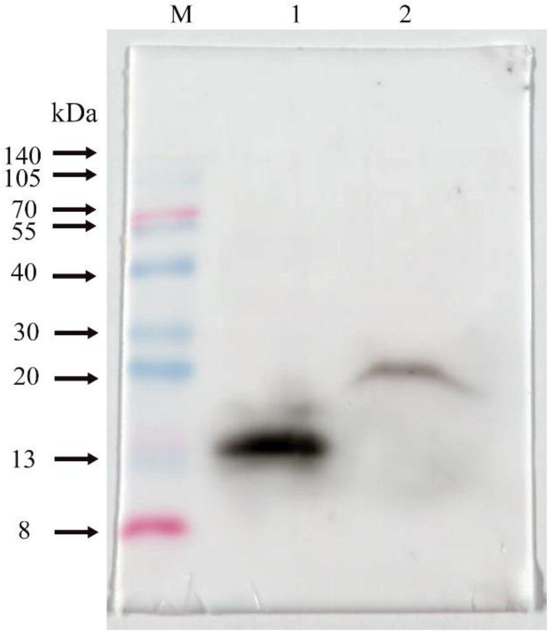

Figure 4 (C) Original Figure (M). Protein molecular weight marker; (1). Purified recombinant protein; (2). Extracted total natural protein.
